# Hypoplastic Left Heart Syndrome: Is There a Role for Fetal Therapy?

**DOI:** 10.3389/fped.2022.944813

**Published:** 2022-07-08

**Authors:** Andreas Tulzer, James C. Huhta, Julian Hochpoechler, Kathrin Holzer, Thomas Karas, David Kielmayer, Gerald Tulzer

**Affiliations:** ^1^Children’s Heart Center Linz, Department of Pediatric Cardiology, Kepler University Hospital, Linz, Austria; ^2^Medical Faculty, Johannes Kepler University Linz, Linz, Austria; ^3^Perinatal Cardiology, St. Joseph Hospital, Tampa, FL, United States

**Keywords:** critical aortic stenosis, hypoplastic left heart, fetal cardiac interventions (FCI), fetal aortic valvuloplasty, maternal hyperoxygenation, restrictive interatrial septum

## Abstract

During fetal life some cardiac defects may lead to diminished left heart growth and to the evolution of a form of hypoplastic left heart syndrome (HLHS). In fetuses with an established HLHS, severe restriction or premature closure of the atrial septum leads to left atrial hypertension and remodeling of the pulmonary vasculature, severely worsening an already poor prognosis. Fetal therapy, including invasive fetal cardiac interventions and non-invasive maternal hyperoxygenation, have been introduced to prevent a possible progression of left heart hypoplasia, improve postnatal outcome, or secure fetal survival. The aim of this review is to cover patient selection and possible hemodynamic effects of fetal cardiac procedures and maternal hyperoxygenation in fetuses with an evolving or established hypoplastic left heart syndrome.

## Introduction

Hypoplasia of the left heart represents a wide spectrum of underdeveloped left ventricular structures, ranging from a borderline left ventricle to a severely hypoplastic left ventricle in the setting of mitral- and aortic atresia. Whereas, with the latter morphology, a postnatal univentricular palliation is inevitable with its known relatively high morbidity and mortality (1–3), a postnatal biventricular approach may be possible in a patient with a borderline left heart or Shone’s complex (4, 5). During gestation, fetal echocardiography may reveal evolution of hypoplastic left heart syndrome (HLHS), making these fetuses potential candidates for fetal therapy, including invasive fetal cardiac interventions and non-invasive maternal hyperoxygenation. These therapies were introduced to promote left heart growth during gestation and to avoid univentricular palliation postnatally. In fetuses with an already established HLHS, patency of the foramen ovale is crucial to enable left atrial drainage. Fetuses with highly restrictive or intact atrial septum are at high risk for mortality postnatally. Fetal diagnostics aim to diagnose fetuses with the need for urgent postnatal care, making those patients suitable candidates for invasive fetal cardiac interventions.

In this review patient selection, hemodynamic effects, and possible benefits of invasive fetal cardiac interventions in patients with an evolving or established HLHS shall be discussed. We will further discuss early results of the prospects of diagnostic and therapeutic use of maternal hyperoxygenation in this group of patients.

## Cardiac Anomalies Associated With Fetal Left Heart Hypoplasia

**FIGURE 1:**
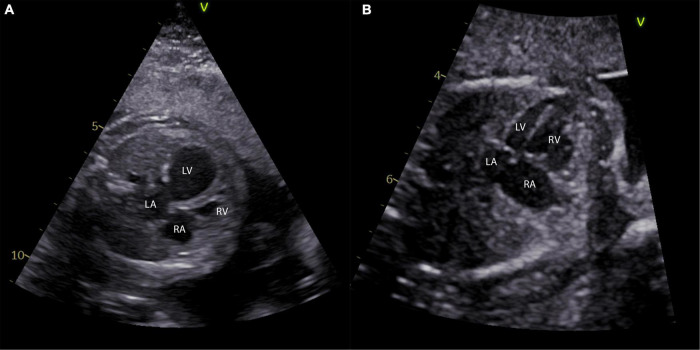
Different phenotypes of left ventricular hypoplasia in the fetus. **(A)** A four-chamber view of a fetus with 24 weeks and critical aortic stenosis is shown with a severely dilated left ventricle. Panel **(B)** shows a four-chamber view of a fetus at 27 weeks with a long and skinny, but apex forming left ventricle. LA, left atrium; LV, left ventricle; RA, right atrium; RV, right ventricle.

**FIGURE 2:**
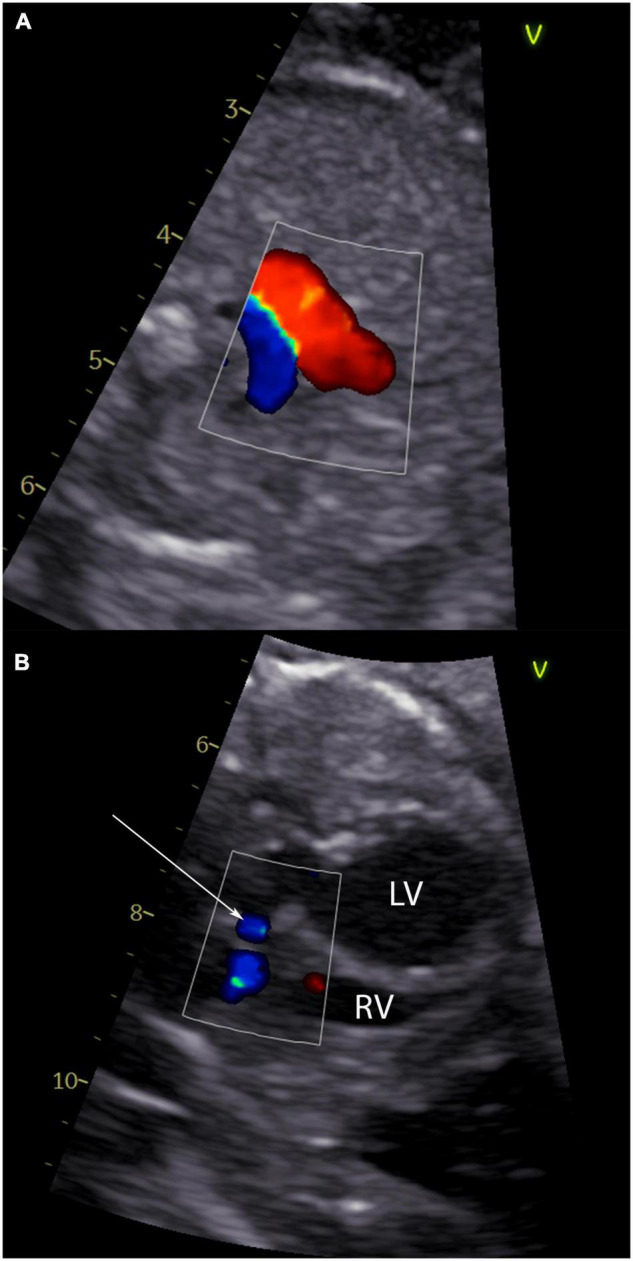
Echocardiographic signs of an evolving hypoplastic left heart syndrome. Panel **(A)** shows a 3-vessel view of a fetus with critical aortic stenosis with antegrade flow in the ductus arteriosus Botalli (red flow) and retrograde flow in the aortic arch (blue flow). In panel **(B)** left to right shunting at the atrial level of the same fetus is shown (arrow).

In fetal echocardiography, hypoplasia of left ventricular structures with mitral patency may present in two different shapes: (1) a poorly contractile, dilated left ventricle associated with critical aortic stenosis, apex or non-apex forming with the presence of endocardial fibroelastosis (4, 6, 7) or (2) a long, skinny apex forming left ventricle with laminar flow across the mitral and aortic valve (Figure 1) (8). The latter morphology may present with or without the presence of an aneurysm of the atrial septum (9) or a persistent left superior vena cava (LSVC) (10). Both phenotypes may present with flow reversal in the aortic arch and bidirectional or left to right shunting at the atrial level, known signs of a possible evolving HLHS, specifically in aortic valve stenosis (Figure 2) (7). Whereas in fetuses with critical aortic stenosis, the left ventricle is impaired in its function and not able to support systemic perfusion due to severe outflow obstruction, it is suspected that reverse flow in the aortic arch in fetuses with a skinny shaped LV, however, may be the consequence of impaired left ventricular loading. Routinely, both phenotypes may be counseled with regard to a possible univentricular palliation postnatally (6, 11–13). However, whereas the natural history in fetal critical aortic stenosis may lead to higher numbers of patients with univentricular outcomes postnatally (14), this may not be the case for patients with other cardiac phenotypes (15).

There have been several investigations with regard to left ventricular growth during fetal life (6, 16, 17). In a recent study by Venardos et al. significant differences in left heart growth according to the underlying phenotype could be found (15). Fetuses with severe left heart hypoplasia, but apex forming left ventricles in the absence of outflow obstruction showed growth at a faster rate compared to aortic stenosis patients, however, Z-scores still decreased during pregnancy (15). Interestingly, there was no difference in left heart growth in the presence of LSVC and also the number of univentricular palliations in fetuses with LSVC was similar (9–10%) (15). Overall, the risk of univentricular palliation in the group of left heart hypoplasia in the absence of aortic stenosis was low, with no patient undergoing univentricular palliation procedures if right to left atrial shunting was present (15).

However, if retrograde aortic arch perfusion or abnormal FO flow is present, difficulties in prenatal counseling of the parents remain, independent of the underlying left ventricular phenotype.

## Invasive Fetal Cardiac Interventions in Critical Aortic Stenosis and Evolving HLHS

**FIGURE 3:**
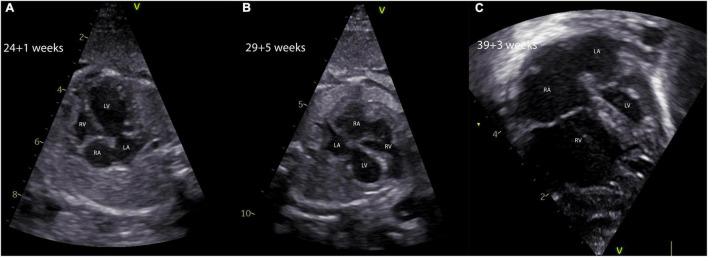
Natural history of a fetus with critical aortic stenosis. The evolution of a hypoplastic left heart syndrome in a fetus with critical aortic stenosis is shown. The fetus was diagnosed at 24 + 1 weeks of gestation with a severely dilated and poorly contracting, but apex forming left ventricle **(A)**. Five weeks later the fetus showed marked signs of left ventricular hypoplasia and severe EFE, the apex being formed by the right ventricle **(B)**. In panel **(C)**, the four-chamber view of the same fetus shows severe left ventricular hypoplasia in terms of a hypoplastic left heart syndrome making a univentricular palliation inevitable. EFE, endocardial fibroelastosis; LA, left atrium; LV, left ventricle; RA, right atrium; RV, right ventricle.

**FIGURE 4:**
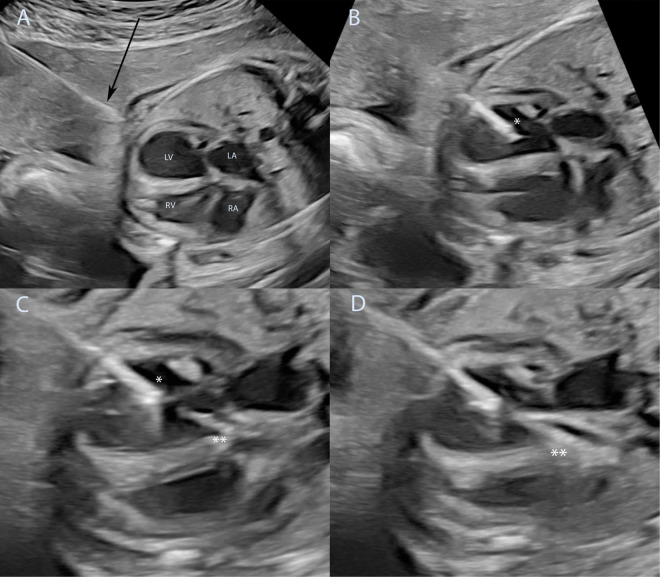
Technique of fetal aortic valvuloplasty performed under ultrasound guidance. Fetal aortic valvuloplasty of a fetus at 25 weeks of gestation with critical aortic stenosis and eHLHS is shown. In panel **(A)** the ultrasound guided puncture of the fetal thoracic wall is shown, the arrow marks the needle just before entering the fetus. The needle has been introduced into the left ventricle **(B)**, asterisk marks the needle tip inside the left ventricle. The guidewire and the balloon catheter have crossed the aortic valve in panel **(C)** and the needle is slightly retracted to allow good positioning of the balloon just before dilation of the aortic valve (^**^). Panel **(D)** shows full insufflation of the balloon catheter (^**^). eHLHS, evolving hypoplastic left heart syndrome; LA, left atrium; LV, left ventricle; RA, right atrium; RV, right ventricle.

Fetuses with critical aortic stenosis are at risk of intrauterine growth arrest of the left ventricle, ultimately leading to HLHS postnatally needing univentricular palliation (Figure 3) (1, 2, 6, 7, 14, 18). Critical aortic stenosis may lead to severe systolic and diastolic dysfunction of the left ventricle with severely increased left ventricular pressures. Endocardial fibroelastosis (EFE) is present in almost all cases of critical aortic stenosis, also negatively affecting left ventricular growth and diastolic function (19), and in severe cases also affecting right ventricular function (20). In the 1990s the first reports were published about patients who underwent fetal aortic valvuloplasty in order to prevent progression of this severe lesion *in utero*, however with results being disappointing initially (21–23). Since the year 2000 several institutions started fetal cardiac intervention programs and reported improving experiences (7, 24–35). Fetal cardiac interventions can be performed with or without general anesthesia of the mother, or with or without separate fetal analgesia or intramuscular relaxation (7, 25, 28, 36). The technique of fetal aortic valvuloplasty is shown in Figure 4. Fetal aortic valvuloplasty may be considered in fetuses showing the following signs: (1) poorly contracting, dilated left ventricle, (2) left to right or bidirectional shunting across the foramen ovale, and (3) retrograde aortic arch flow. However, using only these criteria may also include patients, who would not need prenatal treatment to achieve sufficient left ventricular growth and a biventricular circulation postnatally. In the past two decades the group from Boston, United States and our institution in Linz, Austria tried to optimize inclusion criteria to perform fetal aortic valvuloplasty (26, 27, 31). Ideally, only those fetuses should have prenatal dilation of the aortic valve who would develop HLHS without prenatal treatment. A study by Gardiner et al., however, studied the accuracy of inclusion criteria for fetal intervention published by the group in Boston, showing that up to 30% of fetuses being ideal candidates for fetal aortic valvuloplasty may have achieved a biventricular circulation without prenatal intervention (14). However, this analysis was based on the 2009 criteria (26) and there hasn’t been any evaluation of more recently published criteria for fetal aortic valvuloplasty (27, 31). In 2018, the group in Boston published a decision tree analysis to predict a neonatal biventricular outcome based on LV pressure, LV diastolic diameter, mitral valve size and inflow pattern, and ascending aortic diameter (27). Our group from Linz suggested to use a combination of right to left ventricular length ratio and left ventricular pressure estimates, predicting the circulation type at one year of age with high sensitivity and specificity (31).

So far, the level of evidence to support *in utero* dilation of the aortic valve is still poor as almost all studies only had a retrospective study design, were not randomized, and did not have sufficient control groups. In a recent systematic review by Vorisek et al. (37) no improvement of postnatal biventricular outcome could be found. The same findings were reported by Kovacevic et al. in a retrospective multicenter study with matched cohorts (38). Similar proportions of fetuses showed a biventricular circulation with or without successful fetal aortic valvuloplasty (36 vs. 38%, respectively). However, patients after successful fetal aortic valvuloplasty showed improved survival regardless of final circulation type (38). With regard to postnatal circulation type, institutional bias may affect the results of these multicentric reports (39).

Our institution and the group of the Boston Children’s Hospital frequently observe beneficial hemodynamic effects after aortic valvuloplasty *in utero*. This includes improved antegrade aortic valve flow with antegrade aortic arch perfusion in some cases by the time of discharge, improvement of left ventricular filling with change of the mitral valve Doppler from a short monophasic to a biphasic inflow pattern, and in some cases flow reversal at the atrial level to a bidirectional or right to left shunt (40). These hemodynamic changes, if seen later in gestation, have been shown to be predictive of a biventricular outcome (41). Some patients may have severe aortic regurgitation after fetal aortic valvuloplasty. This, however, is well tolerated by almost all fetuses and usually improves over the remaining pregnancy (42). In fact, a lack of aortic regurgitation after fetal intervention could be a negative predictor of treatment success in our opinion. Valvular remodeling of the aortic valve leads to less severe aortic regurgitation during pregnancy and in some fetuses relevant re-stenosis may occur. This could then negatively influence the overall impact of fetal aortic valvuloplasty if re-stenosis occurs too soon.

Fetal aortic valvuloplasty does not only have the potential to positively affect hemodynamics during pregnancy, but also survival of fetuses. A subset of patients with critical aortic stenosis will develop non-immune hydrops due to impaired right ventricular filling and increased venous pressure. These patients are likely to die during pregnancy or right after birth (43, 44). However, we were able to show that a timely performed FAV in these fetuses led to the resolution of hydrops in two thirds of patients and 50% of the successfully treated patients achieved a postnatal biventricular circulation, all alive at last follow up (30).

The promise of affecting hemodynamics positively in fetuses with critical aortic stenosis led to the initiation of several fetal cardiac intervention programs worldwide. However, as numbers of fetal aortic valvuloplasties performed worldwide are still increasing, center volumes often remain small compared to a few high-volume centers. It has been shown that fetal aortic valvuloplasty may lead to a survival benefit in patients with critical aortic stenosis and evolving hypoplastic left heart syndrome, if procedure related mortality does not exceed 12% (45). It has also been shown for high volume centers, that procedural success rates and procedure related mortality can be improved after a learning curve (26–28, 31), however, smaller centers may not overcome their learning curve due to the low rate of performed procedures with a persisting mortality above 10–12%, compared to 4–5% at our institution or at Boston Children’s Hospital (27, 31).

There have been concerns that fetal aortic valvuloplasty could be a risk factor for preterm deliveries (38). It is important to state that in our experience no association between fetal cardiac interventions and preterm deliveries could be found (31). Increased rates of preterm deliveries of a previous study (38) could be explained by local treatment strategies by the means of preterm cesarean sections. However, in our practice term deliveries (39–40 weeks gestation) are warranted to avoid the known complications of prematurity.

In summary, there is increasing evidence that in selected cases, fetal aortic valvuloplasty has benefits compared to expectant management. Significant hemodynamic improvements can be achieved in some fetuses up to a point where no postnatal therapy is needed (31). However, some will not show persistent benefits following this procedure, and hence will have a low probability of a postnatal biventricular outcome. Fetal aortic valvuloplasty may positively influence overall survival if procedure related mortality does not exceed 12% (45). Ideally, procedure related mortality should be zero or at least less than 5%. In our opinion this can only be achieved with an adequate rate of performed procedures, which is a strong argument for centralization (46). In some fetuses with hydrops, fetal aortic valvuloplasty may even be lifesaving. There are still no ideal criteria when to perform invasive prenatal treatment in these patients. Hence, there is still work to do to further improve selection criteria, improve technical equipment and imaging as well as postnatal treatment strategies for these challenging patients. Prospective data is urgently needed to provide solid evidence about the beneficial effects.

## Fetal Cardiac Interventions in Fetuses With HLHS and Highly Restrictive or Intact Interatrial Septum

**FIGURE 5:**
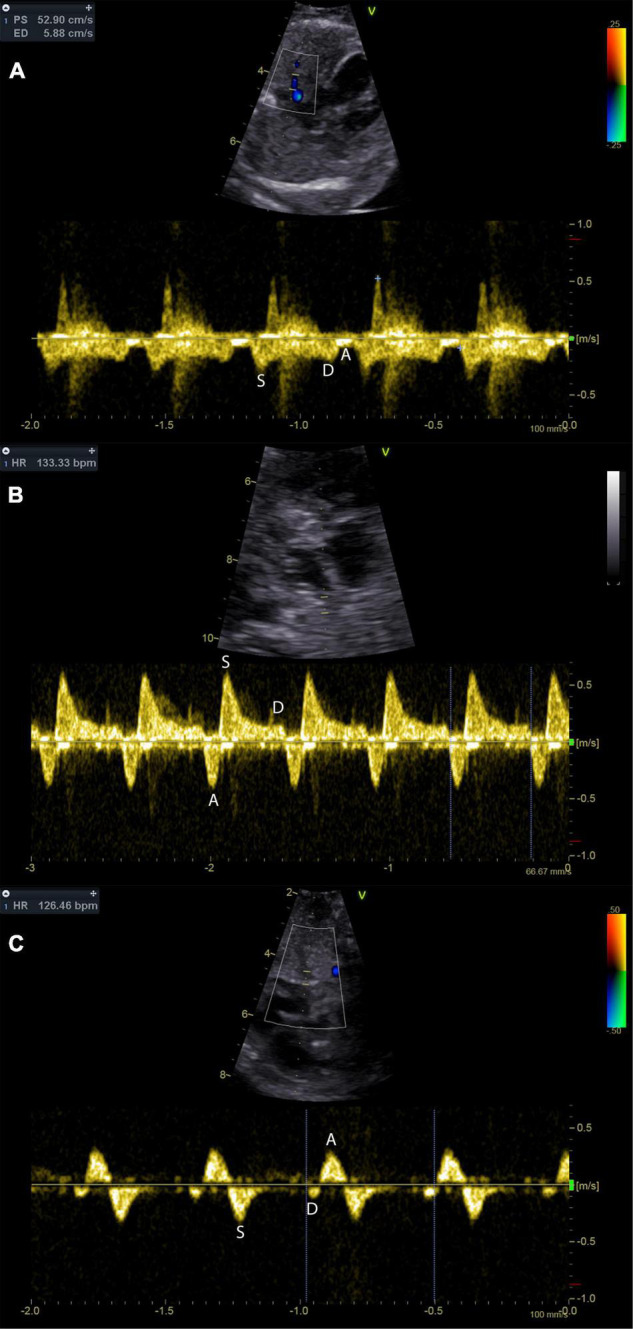
Pulmonary vein Doppler patterns in the hypoplastic left heart with incremental restriction of the interatrial communication. Panel **(A)** shows a fetus with CAS and an unrestrictive interatrial communication; note that there is reduced flow during atrial systole (A-wave). Panel **(B)** shows a fetus with HLHS and restrictive, but patent atrial septum. Prominent A-wave flow reversal during atrial systole may be indicative of relevant restriction. Panel **(C)** shows the pulmonary venous flow pattern in a fetus with HLHS and intact atrial septum. Diastolic pulmonary vein flow is almost missing.

**FIGURE 6:**
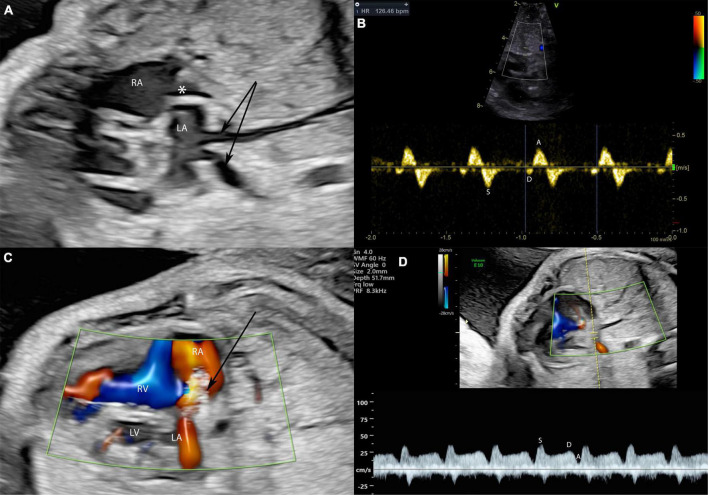
A fetus at 28 weeks gestation with HLHS and intact atrial septum undergoing FCI. Stenting of the interatrial septum in a fetus with intact atrial septum and HLHS is shown. In panel **(A)** severely dilated pulmonary veins (arrows) indicate severe left atrial hypertension. The asterisk marks a thickened and into the right atrium bowing atrial septum. In panel **(B)** severe left atrial hypertension is indicated by severely abnormal pulmonary vein Doppler with almost missing diastolic flow and severe A-wave reversal during atrial systole. The arrow in panel **(C)** marks the placed atrial septum stent with good flow through the stent indicated by the red color Doppler signal. Note that the pulmonary vein Doppler in panel **(D)** is almost normalized in the same patient directly after stent placement with good diastolic flow and no presence of A-Wave reversal.

Severe restriction or an intact atrial septum significantly aggravates the overall prognosis in fetuses with an established HLHS (47–49). In this group of patients, especially in mitral or aortic atresia, the foramen ovale is the only exit of pulmonary venous blood flow in the absence of decompressing veins. If there is severe restriction or even closure of the interatrial communication, left atrial and consequently pulmonary venous hypertension results in markedly pathological remodeling of pulmonary vasculature during gestation, including pulmonary arterial hypertrophy, arterialization of pulmonary veins and intrapulmonary dilatation of lymphatic vessels (49, 50). Figure 5 shows different pulmonary vein Doppler tracings with increasing restriction at the atrial level. Figure 6 shows a fetus at 28 weeks gestation with an intact atrial septum undergoing FCI. Moreover, patients born with this physiology need urgent emergency postnatal care, or even EXIT (ex utero intrapartum treatment) procedures to stabilize hemodynamics directly after birth. However, despite improved postnatal care for this critically ill subset of patients, the overall outcome is still poor with mortality rates of up to 100% (51, 52). Invasive fetal cardiac interventions on the atrial septum have been introduced to enable unrestrictive interatrial blood flow to prevent or even reverse pathological remodeling of the pulmonary vasculature and to enable stable hemodynamics directly after birth (53, 54). In 2004 and 2008 the first results of fetal atrial septoplasty were published by Marshall et al., showing only poor hemodynamic effects with persistent pulmonary venous flow reversal and a remaining high mortality (53, 54). Improvements in hemodynamics could only be achieved if ASDs bigger than 3mm were created, however, this was achieved only in 30% of patients (53). Furthermore, a relevant proportion of patients still needed emergent postnatal interventions due to severe hypoxemia. Therefore, in order to achieve sustained interatrial shunting and to avoid recoiling of the interatrial septum, modifications to fetal balloon septoplasty, laser guided atrial septostomy and stenting of the interatrial septum were proposed (55–58). So far, there are only a few case reports on laser guided atrial septostomy, whereas the experience with intrauterine stenting of the atrial septum is increasing. First experiences of atrial septum stenting were published by the group in Toronto, Canada, showing that intrauterine stenting is technically feasible (57), allows vaginal delivery of this critically ill subset of patients and improved outcomes (56). To create adequate interatrial communications, stents with 2.75-3.00 mm diameter are used and dilated up to 3.6 mm (56). After fetal stenting, endocardial cell proliferation could be a concerning factor and may lead to re-obstruction of the interatrial communication (57). Moreover, alveolar lung pathology may not be fully reversed (56) and there are still technical challenges making this procedure very difficult to perform (59). Therefore, careful evaluation is needed with regard to which patients should undergo *in utero* therapy. At our institution fetuses with HLHS and restrictive atrial septum with a pulmonary vein Doppler forward/reverse VTI ratio of less than 3 and all fetuses with an intact atrial septum are considered suitable candidates for atrial septum stenting. One of the challenges during this procedure is sometimes poor visualization of the septum and stent due to secondary distortion of the interatrial septum as the cannula is passed through (59). In the biggest series on invasive fetal cardiac interventions on the atrial septum in this group of patients, there were no differences found in discharge survival between all FCI and non-FCI patients (34 vs. 36%) (60). In the same study a higher 1 year survival was found in FCI survivors with an unrestrictive FO at birth compared to patients not undergoing invasive fetal cardiac therapy (59 vs. 19%, *p* = 0.03) (60). However, the results of this study may be biased by the fact that data was generated by multiple institutions, there was no standardized postnatal approach in all institutions, and in 25% of post-discharge patients outcome data was missing. Also the authors concluded that FCI should be continued, but concentrated to selected institutions to concentrate experience and standardize fetal and postnatal care (60).

If early third trimester interventions are not attempted, stenting of the interatrial septum just before birth may still be a helpful alternative to avoid postnatal EXIT procedures, as shown by our institution (61). This method may be more effective than early third trimester stenting, as there is less time for endocardial cell proliferation, possibly leading to important re-obstruction at term. However, the main objective to perform stenting is to reverse or prevent irreversible damage to improve the pulmonary vasculature and to prevent alveolar damage to the lungs. At this point it is unclear whether this intervention can achieve both, especially if late stenting is performed just before birth.

In summary, stenting of the interatrial septum seems to be promising and may positively affect outcome in these critically ill patients compared to the natural course. As this technique is still challenging, centralization should be attempted to gain more experience in this field and to standardize treatment protocols.

## Maternal Hyperoxygenation in Fetuses With Left Heart Hypoplasia

In the absence of critical aortic stenosis, there are also other congenital cardiac anomalies leading to significant hypoplasia of left sided structures, being summarized under the diagnosis of Shone’s complex (4, 5). In some cases, there is significant underdevelopment of the mitral valve, with a left ventricle still apex forming, but ultimately not suitable for a biventricular approach postnatally. During the past decade, maternal hyperoxygenation (MH) was introduced and studied with regard to (1) promoting left heart growth during gestation to avoid a postnatal univentricular palliation, (2) the impact of MH on fetuses with left heart hypoplasia and atrial septal aneurysm, and (3) to diagnose those with an already established HLHS with regard to relevant restriction across the interatrial communication with the potential need of urgent emergency postnatal care (16, 62–66).

## Maternal Hyperoxygenation Therapy to Stimulate Left Heart Growth

The principle of MH is based upon oxygen induced reduction of fetal pulmonary vascular resistance (PVR). Response of fetal pulmonary vasculature to supplemental oxygen has been shown to occur by the end of 26 weeks of gestation (67). Reduced fetal PVR should lead to increased pulmonary arterial blood flow, and thus increased pulmonary venous return. This increased left atrial volume should therefore stimulate mitral valve, left ventricular, and aortic valve growth. In 2010, Kohl was the first to describe positive effects of chronic intermittent MH on left heart growth (65). There were significant increases in pulmonary venous mean flow velocities as well as increases in mitral valve late diastolic flow velocities (65). One specific finding was that fetuses with lower left heart dimensions had more favorable growth of left sided structures compared to fetuses closer to the normal range. Another finding of the Kohl study was that there was no or little effect on left cardiac structures seen in fetuses with malformations like LSVC, otherwise obstructed or stenotic AV-valves, or obstructions of left ventricular outflow including aortic valve stenosis (65). However, there were no control cases, neither historic nor prospective, to compare the changes of left heart dimensions. A possible implication of this study was that these potential beneficial effects could also be used in addition to fetuses undergoing invasive fetal aortic valvuloplasty, to improve recovery of left ventricular function and growth by improved left heart loading (65). In 2016, the group from Houston, Texas, performed a pilot study on left heart dimensional growth after chronic MH using historic controls (63). The initial findings of this study were not as significant as described in the Kohl study. There was increased aortic flow compared to baseline during acute MH testing, but there was no significant difference with regard to aortic valve growth compared to the historic control cases, arguably being attributable to the small cohort size (63). Nevertheless, an important question could have been answered by this study: what is the optimal duration to perform MH per day to improve left heart growth? In patients with a mean duration of more than 9 hours per day, better aortic annular growth was observed compared to the historical control group. Nevertheless, the same mean duration did not lead to significantly improved mitral valve growth (63). Both studies mentioned above did not report any severe complications, neither for the fetus or neonate, nor for the mother (63, 65). However, there have also been concerns with regard to fetal complications in patients undergoing chronic intermittent hyperoxygenation. One study reported that chronic MH may be associated with growth restriction (63), whereas another group had concerns regarding fetal brain and placental development (16). In animal models using fetal sheep it could also be shown that hyperoxygenation led to reduced cerebral perfusion resulting in significantly decreased cerebral glucose delivery and consumption (68). Other fetal complications like constriction of the arterial duct or retinopathy may also be considered but were not reported in human studies on chronic MH. Due to the lack of data in the field of chronic MH and possibly associated fetal complications, the recommendation of a recent review was that studies on MH should be curtailed (69).

Altogether, as summarized previously, chronic MH may have beneficial effects on left heart growth (70). In presence of left to right shunting at the atrial level these effects may be limited only to those patients with a somewhat restrictive interatrial communication, but there is no data yet to support this hypothesis.

Chronic MH should be initiated at about 50% FiO2 for at least 9 h per day with only few interruptions to avoid repeated changes in fetal hemodynamics (16, 70).

Prospectively controlled studies should be performed to support the previously reported effects on left heart growth and to further study possible fetal complications.

## Acute Hyperoxygenation Testing in Fetuses With Left Heart Hypoplasia

The hemodynamic effects of MH have been shown to be useful in prenatal diagnosis and counseling. Acute MH in fetuses with left heart hypoplasia was specifically studied in patients with HLHS and restrictive or intact atrial septum (62, 66, 71, 72). Severe restriction or an intact atrial septum significantly aggravates the overall prognosis in fetuses with hypoplastic left heart syndrome as described above. It has been demonstrated that acute MH for 10 min with 60% FiO_2_ reliably identified fetuses with pulmonary vascular damage compared to fetuses with HLHS and unrestrictive interatrial flow (62, 66). The group from the Children’s Hospital of Philadelphia demonstrated, that acute MH in fetuses with unrestrictive interatrial communications led to a significant reduction in pulmonary artery PI (62). In contrast, fetuses with HLHS and restrictive atrial septum (RAS) did not show a significant change in the pulmonary artery PI (<10% decrease) in response to acute MH, thus being a possible reliable predictor for urgent postnatal intervention (62). Similar findings were reported in a study by Enzensberger et al., also showing that there was no response to acute MH in fetuses with HLHS and RAS (66). Mardy et al. (72), however, investigated the effect of acute MH on pulmonary venous return analyzing the pulmonary vein prograde/retrograde VTI ratio (48, 73–75) with regard to emergent postnatal intervention. Different pulmonary vein flow patterns with increasing restriction of the atrial septum are shown in Figure 5. Emergent postnatal intervention could be predicted with 100% sensitivity and 100% specificity by using the following classification: Fetuses with a baseline Pvein VTI ratio <3 were ascribed to the emergent intervention group as well as fetuses with a Pvein VTI ratio between 3 and 7 with a decrease from baseline to MH (72). The branch pulmonary artery PI showed less sensitivity and specificity in the same study (72).

However, acute MH may also improve accurate diagnosis of left heart hypoplasia in fetuses with atrial septal aneurysms leading to impaired left ventricular filling (9). In a case series of 12 fetuses with apparent left ventricular hypoplasia due to impaired LV loading by aneurysm of the atrial septum, acute MH demonstrated significant hemodynamic effects: after 10 minutes of acute MH, increased pulmonary venous return led to a significant reduction in atrial septal excursion leading to improved LV loading, and consequently changed the flow pattern in the aortic arch in all participating fetuses from retrograde to antegrade or bidirectional (9). Importantly, all studies on acute MH testing did not report any adverse reactions, neither fetal nor maternal.

In summary, maternal hyperoxygenation offers possible adjuncts to current diagnostic and treatment strategies. Especially in fetuses with HLHS and restrictive interatrial septum, acute MH has the potential to identify those with the need of high urgent postnatal care and may help in the decision making, if invasive fetal therapy should be performed. Currently, acute MH is not performed on a routine basis across many centers. However, this diagnostic element can be implemented easily. Moreover, chronic MH may improve left heart development. Using chronic MH could show further benefits in fetuses after fetal aortic valvuloplasty. However, there is yet no data to support this and prospective controlled research trials should be performed to increase the currently low level of evidence in this field and the safety of this treatment, which is still under investigation.

## Conclusion and Outlook

There has been tremendous progress in invasive fetal treatment in the past two decades and there is increasing evidence that invasive fetal treatment has survival benefits compared to expectant management. Invasive fetal therapy is only the first step in the treatment of this critically ill subset of patients and adequate postnatal therapy is needed to sustain and secure the impact of the performed prenatal procedure. Until now, fetal cardiac interventions haven’t been the focus of the industry and hence there has been only little to no evolution in instruments used during these procedures. Technical advances are warranted to improve outcomes and procedural security. Invasive fetal cardiac interventions are no routine procedures at this moment and still carry a significant risk of fetal demise if not performed in a high-volume center. Therefore, invasive fetal treatment should be centralized to a few institutions, which have overcome their learning curve and are able to perform these procedures on a regular basis.

Chronic maternal hyperoxygenation may be a non-invasive treatment-alternative to promote left heart growth in fetuses with left heart hypoplasia, but the evidence on this topic is limited and safety regarding neurodevelopmental outcome is yet unknown.

Nonetheless, acute maternal hyperoxygenation has been shown to be of value with regard to prenatal diagnosis and counseling.

Prospectively controlled studies are warranted for both, invasive and non-invasive fetal therapy, to improve the level of evidence in this small field of perinatal and pediatric cardiology.

## Author Contributions

GT, JHu, and AT were the main responsible authors with regard to drafting and critical revision. All authors made contributions to the acquisition and analysis of literature for the manuscript, agreed to be accountable for all aspects of the work, and provided approval upon submission of the manuscript.

## Conflict of Interest

The authors declare that the research was conducted in the absence of any commercial or financial relationships that could be construed as a potential conflict of interest.

## Publisher’s Note

All claims expressed in this article are solely those of the authors and do not necessarily represent those of their affiliated organizations, or those of the publisher, the editors and the reviewers. Any product that may be evaluated in this article, or claim that may be made by its manufacturer, is not guaranteed or endorsed by the publisher.

## References

[1] Newburger JW Sleeper LA Frommelt PC Pearson GD Mahle WT Chen S Transplantation-free survival and interventions at 3 years in the single ventricle reconstruction trial. *Circulation.* (2014) 129:2013–20. 24705119 10.1161/CIRCULATIONAHA.113.006191 PMC4029928

[2] Ghanayem NS Allen KR Tabbutt S Atz AM Clabby ML Cooper DS Interstage mortality after the Norwood procedure: results of the multicenter single ventricle reconstruction trial. *J Thorac Cardiovasc Surg.* (2012) 144:896–906. 22795436 10.1016/j.jtcvs.2012.05.020 PMC3985484

[3] Newburger JW Sleeper LA Gaynor JW Hollenbeck-Pringle D Frommelt PC Li JS Transplant-free survival and interventions at 6 years in the SVR trial. *Circulation.* (2018) 137:2246–53. 29437119 10.1161/CIRCULATIONAHA.117.029375 PMC5963989

[4] Hickey EJ Caldarone CA McCrindle BW. Left ventricular hypoplasia: a spectrum of disease involving the left ventricular outflow tract, aortic valve, and aorta. *J Am Coll Cardiol.* (2012) 59(1 Suppl.):S43–54. 22192721 10.1016/j.jacc.2011.04.046

[5] Akintürk H Valeske K Müller M Bauer J Thul J Michel-Behnke I Biventricular repair in children with hypoplastic left heart complex. *Thorac Cardiovasc Surg.* (2007) 55(S 1):V_6.

[6] Hornberger LK Sanders SP Rein AJ Spevak PJ Parness IA Colan SD. Left heart obstructive lesions and left ventricular growth in the midtrimester fetus. A longitudinal study. *Circulation.* (1995) 92:1531–8. 10.1161/01.cir.92.6.1531 7664437

[7] Mäkikallio K McElhinney DB Levine JC Marx GR Colan SD Marshall AC Fetal aortic valve stenosis and the evolution of hypoplastic left heart syndrome: patient selection for fetal intervention. *Circulation.* (2006) 113:1401–5. 10.1161/CIRCULATIONAHA.105.588194 16534003

[8] Corno AF. Borderline left ventricle. *Eur J Cardiothorac Surg.* (2005) 27:67–73. 15621473 10.1016/j.ejcts.2004.10.034

[9] Channing A Szwast A Natarajan S Degenhardt K Tian Z Rychik J. Maternal hyperoxygenation improves left heart filling in fetuses with atrial septal aneurysm causing impediment to left ventricular inflow. *Ultrasound Obstet Gynecol.* (2015) 45:664–9. 25296951 10.1002/uog.14688

[10] Liu X He Y Tian Z Rychik J. Persistent left superior vena cava connected to the coronary sinus in the fetus: effects on cardiac structure and flow dynamics. *Pediatr Cardiol.* (2016) 37:1085–90. 10.1007/s00246-016-1395-6 27084383

[11] Allan LD Sharland G Tynan MJ. The natural history of the hypoplastic left heart syndrome. *Int J Cardiol.* (1989) 25:341–3. 2613383 10.1016/0167-5273(89)90226-x

[12] Sharland GK Chita SK Fagg NL Anderson RH Tynan M Cook AC Left ventricular dysfunction in the fetus: relation to aortic valve anomalies and endocardial fibroelastosis. *Br Heart J.* (1991) 66:419–24. 10.1136/hrt.66.6.419 1837727 PMC1024814

[13] Simpson JM Sharland GK. Natural history and outcome of aortic stenosis diagnosed prenatally. *Heart.* (1997) 77:205–10. 9093035 10.1136/hrt.77.3.205 PMC484683

[14] Gardiner HM Kovacevic A Tulzer G Sarkola T Herberg U Dangel J Natural history of 107 cases of fetal aortic stenosis from a European multicenter retrospective study. *Ultrasound Obstet Gynecol.* (2016) 48:373–81. 10.1002/uog.15876 26843026

[15] Venardos A Colquitt J Morris SA. Fetal growth of left-sided structures and postnatal surgical outcomes in the ‘borderline’ left heart varies by cardiac phenotype. *Ultrasound Obstet Gynecol.* (2021) 59:642–650. 10.1002/uog.23689 33998097

[16] Edwards LA Lara DA Sanz Cortes M Hunter JV Andreas S Nguyen MJ Chronic maternal hyperoxygenation and effect on cerebral and placental vasoregulation and neurodevelopment in fetuses with left heart hypoplasia. *Fetal Diagn Ther.* (2019) 46:45–57. 10.1159/000489123 30223262

[17] Emani SM del Nido PJ. Strategies to maintain biventricular circulation in patients with high-risk anatomy. *Semin Thorac Cardiovasc Surg Pediatr Card Surg Annu.* (2013) 16:37–42. 10.1053/j.pcsu.2013.01.003 23561816

[18] Axt-Fliedner R Kreiselmaier P Schwarze A Krapp M Gembruch U. Development of hypoplastic left heart syndrome after diagnosis of aortic stenosis in the first trimester by early echocardiography. *Ultrasound Obstet Gynecol.* (2006) 28:106–9. 10.1002/uog.2824 16795135

[19] McElhinney DB Vogel M Benson CB Marshall AC Wilkins-Haug LE Silva V Assessment of left ventricular endocardial fibroelastosis in fetuses with aortic stenosis and evolving hypoplastic left heart syndrome. *Am J Cardiol.* (2010) 106:1792–7. 21126622 10.1016/j.amjcard.2010.08.022

[20] Graupner O Enzensberger C Wieg L Degenhardt J Wolter A Khalil M endocardial fibroelastosis of the left ventricle affects right ventricular performance in fetuses with hypoplastic left heart syndrome: a prospective study using M-mode, PW- and tissue Doppler techniques. *Ultraschall Med.* (2018) 39:413–21. 10.1055/s-0043-111590 28683514

[21] Kohl T Sharland G Allan LD Gembruch U Chaoui R Lopes LM World experience of percutaneous ultrasound-guided balloon valvuloplasty in human fetuses with severe aortic valve obstruction. *Am J Cardiol.* (2000) 85:1230–3. 10.1016/s0002-9149(00)00733-5 10802006

[22] Maxwell D Allan L Tynan MJ. Balloon dilatation of the aortic valve in the fetus: a report of two cases. *Br Heart J.* (1991) 65:256–8. 2039669 10.1136/hrt.65.5.256 PMC1024626

[23] Lopes LM Cha SC Kajita LJ Aiello VD Jatene A Zugaib M. Balloon dilatation of the aortic valve in the fetus. A case report. *Fetal Diagn Ther.* (1996) 11:296–300. 8823612 10.1159/000264318

[24] Freud LR McElhinney DB Marshall AC Marx GR Friedman KG del Nido PJ Fetal aortic valvuloplasty for evolving hypoplastic left heart syndrome: postnatal outcomes of the first 100 patients. *Circulation.* (2014) 130:638–45. 10.1161/CIRCULATIONAHA.114.009032 25052401 PMC4299861

[25] Tworetzky W Wilkins-Haug L Jennings RW van der Velde ME Marshall AC Marx GR Balloon dilation of severe aortic stenosis in the fetus: potential for prevention of hypoplastic left heart syndrome: candidate selection, technique, and results of successful intervention. *Circulation.* (2004) 110:2125–31. 10.1161/01.CIR.0000144357.29279.54 15466631

[26] McElhinney DB Marshall AC Wilkins-Haug LE Brown DW Benson CB Silva V Predictors of technical success and postnatal biventricular outcome after in utero aortic valvuloplasty for aortic stenosis with evolving hypoplastic left heart syndrome. *Circulation.* (2009) 120:1482–90. 10.1161/CIRCULATIONAHA.109.848994 19786635 PMC4235336

[27] Friedman KG Sleeper LA Freud LR Marshall AC Godfrey ME Drogosz M Improved technical success, postnatal outcome and refined predictors of outcome for fetal aortic valvuloplasty. *Ultrasound Obstet Gynecol.* (2018) 52:212–20. 28543953 10.1002/uog.17530

[28] Arzt W Wertaschnigg D Veit I Klement F Gitter R Tulzer G. Intrauterine aortic valvuloplasty in fetuses with critical aortic stenosis: experience and results of 24 procedures. *Ultrasound Obstet Gynecol.* (2011) 37:689–95. 21229549 10.1002/uog.8927

[29] Debska M Kolesnik A Rebizant B Sekowska A Grzyb A Chaberek K Fetal cardiac interventions-polish experience from ‘zero’ to the third world largest program. *J Clin Med.* (2020) 9:2888. 10.3390/jcm9092888 32906670 PMC7576494

[30] Tulzer A Arzt W Tulzer G. Fetal aortic valvuloplasty may rescue fetuses with critical aortic stenosis and hydrops. *Ultrasound Obstet Gynecol.* (2021) 57:119–25. 10.1002/uog.22138 32621387

[31] Tulzer A Arzt W Gitter R Sames-Dolzer E Kreuzer M Mair R Valvuloplasty in 103 fetuses with critical aortic stenosis: outcome and new predictors for postnatal circulation. *Ultrasound Obstet Gynecol.* (2021) 59:633–641. 10.1002/uog.24792 34605096 PMC9324970

[32] Moon-Grady AJ Morris SA Belfort M Chmait R Dangel J Devlieger R International fetal cardiac intervention registry: a worldwide collaborative description and preliminary outcomes. *J Am Coll Cardiol.* (2015) 66:388–99. 10.1016/j.jacc.2015.05.037 26205597

[33] Galindo A Gómez-Montes E Gómez O Bennasar M Crispi F Herraiz I Fetal aortic valvuloplasty: experience and results of two tertiary centers in Spain. *Fetal Diagn Ther.* (2017) 42:262–70. 10.1159/000460247 28384638

[34] Pedra SRFF Peralta CFA Crema L Jatene IB da Costa RN Pedra CAC. Fetal interventions for congenital heart disease in Brazil. *Pediatr Cardiol.* (2014) 35:399–405. 24030590 10.1007/s00246-013-0792-3

[35] Patel ND Nageotte S Ing FF Armstrong AK Chmait R Detterich JA Procedural, pregnancy, and short-term outcomes after fetal aortic valvuloplasty. *Catheter Cardiovasc Interv.* (2020) 96:626–32. 10.1002/ccd.28846 32216096

[36] Tulzer G Arzt W Franklin RCG Loughna PV Mair R Gardiner HM. Fetal pulmonary valvuloplasty for critical pulmonary stenosis or atresia with intact septum. *Lancet.* (2002) 360:1567–8. 12443597 10.1016/S0140-6736(02)11531-5

[37] Vorisek CN Zurakowski D Tamayo A Axt-Fliedner R Siepmann T Friehs I. Postnatal circulation in patients with aortic stenosis undergoing fetal aortic valvuloplasty: systematic review and meta-analysis. *Ultrasound Obstet Gynecol.* (2021) 59:576–584. 10.1002/uog.24807 34726817

[38] Kovacevic A Öhman A Tulzer G Herberg U Dangel J Carvalho JS Fetal hemodynamic response to aortic valvuloplasty and postnatal outcome: a European multicenter study. *Ultrasound Obstet Gynecol.* (2018) 52:221–9. 10.1002/uog.18913 28976617

[39] Kovacevic A Roughton M Mellander M Öhman A Tulzer G Dangel J Fetal aortic valvuloplasty: investigating institutional bias in surgical decision-making. *Ultrasound Obstet Gynecol.* (2014) 44:538–44. 10.1002/uog.13447 24975801

[40] Prosnitz AR Drogosz M Marshall AC Wilkins-Haug LE Benson CB Sleeper LA Early hemodynamic changes after fetal aortic stenosis valvuloplasty predict biventricular circulation at birth. *Prenat Diagn.* (2018) 38:286–92. 10.1002/pd.5232 29436717 PMC6986395

[41] Beattie MJ Friedman KG Sleeper LA Lu M Drogosz M Callahan R Late gestation predictors of a postnatal biventricular circulation after fetal aortic valvuloplasty. *Prenat Diagn.* (2021) 41:479–85. 10.1002/pd.5885 33462820 PMC11771853

[42] Marshall AC Tworetzky W Bergersen L McElhinney DB Benson CB Jennings RW Aortic valvuloplasty in the fetus: technical characteristics of successful balloon dilation. *J Pediatr.* (2005) 147:535–9. 10.1016/j.jpeds.2005.04.055 16227042

[43] Vogel M McElhinney DB Wilkins-Haug LE Marshall AC Benson CB Juraszek AL Aortic stenosis and severe mitral regurgitation in the fetus resulting in giant left atrium and hydrops: pathophysiology, outcomes, and preliminary experience with pre-natal cardiac intervention. *J Am Coll Cardiol.* (2011) 57:348–55. 10.1016/j.jacc.2010.08.636 21232673

[44] Mallmann MR Herberg U Gottschalk I Strizek B Hellmund A Geipel A Fetal cardiac intervention in critical aortic stenosis with severe mitral regurgitation, severe left atrial enlargement, and restrictive foramen ovale. *Fetal Diagn Ther.* (2019) 47:440–447. 10.1159/000502840 31593939

[45] Pickard SS Wong JB Bucholz EM Newburger JW Tworetzky W Lafranchi T Fetal aortic valvuloplasty for evolving hypoplastic left heart syndrome: a decision analysis. *Circ Cardiovasc Qual Outcomes.* (2020) 13:e006127. 32252549 10.1161/CIRCOUTCOMES.119.006127 PMC7737668

[46] Kang SL Jaeggi E Ryan G Chaturvedi RR. An overview of contemporary outcomes in fetal cardiac intervention: a case for high-volume superspecialization? *Pediatr Cardiol.* (2020) 41:479–85. 10.1007/s00246-020-02294-2 32198586

[47] Jadczak A Respondek-Liberska M Sokołowski Ł Chrzanowski J Rizzo G Araujo E Jr Hypoplastic left heart syndrome with prenatally diagnosed foramen ovale restriction: diagnosis, management and outcome. *J Matern Fetal Neonatal Med.* (2022) 35:291–8. 31986935 10.1080/14767058.2020.1716717

[48] Divanović A Hor K Cnota J Hirsch R Kinsel-Ziter M Michelfelder E. Prediction and perinatal management of severely restrictive atrial septum in fetuses with critical left heart obstruction: clinical experience using pulmonary venous Doppler analysis. *J Thorac Cardiovasc Surg.* (2011) 141:988–94. 10.1016/j.jtcvs.2010.09.043 21130471

[49] Graziano JN Heidelberger KP Ensing GJ Gomez CA Ludomirsky A. The influence of a restrictive atrial septal defect on pulmonary vascular morphology in patients with hypoplastic left heart syndrome. *Pediatr Cardiol.* (2002) 23:146–51. 10.1007/s00246-001-0038-7 11889524

[50] Rychik J Rome JJ Collins MH DeCampli WM Spray TL. The hypoplastic left heart syndrome with intact atrial septum: atrial morphology, pulmonary vascular histopathology and outcome. *J Am Coll Cardiol.* (1999) 34:554–60. 10440172 10.1016/s0735-1097(99)00225-9

[51] Arai S Fujii Y Kotani Y Kuroko Y Kasahara S Sano S. Surgical outcome of hypoplastic left heart syndrome with intact atrial septum. *Asian Cardiovasc Thorac Ann.* (2015) 23:1034–8. 26405018 10.1177/0218492315606581

[52] Sathanandam SK Philip R Gamboa D Van Bergen A Ilbawi MN Knott-Craig C Management of hypoplastic left heart syndrome with intact atrial septum: a two-centre experience. *Cardiol Young.* (2016) 26:1072–81. 10.1017/S1047951115001791 26346529

[53] Marshall AC Levine J Morash D Silva V Lock JE Benson CB Results of in utero atrial septoplasty in fetuses with hypoplastic left heart syndrome. *Prenat Diagn.* (2008) 28:1023–8. 18925607 10.1002/pd.2114

[54] Marshall AC van der Velde ME Tworetzky W Gomez CA Wilkins-Haug L Benson CB Creation of an atrial septal defect in utero for fetuses with hypoplastic left heart syndrome and intact or highly restrictive atrial septum. *Circulation.* (2004) 110:253–8. 15226215 10.1161/01.CIR.0000135471.17922.17

[55] Quintero RA Huhta J Suh E Chmait R Romero R Angel J. In utero cardiac fetal surgery: laser atrial septotomy in the treatment of hypoplastic left heart syndrome with intact atrial septum. *Am J Obstet Gynecol.* (2005) 193:1424–8. 10.1016/j.ajog.2005.02.126 16202736

[56] Chaturvedi RR Ryan G Seed M van Arsdell G Jaeggi ET. Fetal stenting of the atrial septum: technique and initial results in cardiac lesions with left atrial hypertension. *Int J Cardiol.* (2013) 168:2029–36. 10.1016/j.ijcard.2013.01.173 23481911

[57] Schmidt M Jaeggi E Ryan G Hyldebrandt J Lilly J Peirone A Percutaneous ultrasound-guided stenting of the atrial septum in fetal sheep. *Ultrasound Obstet Gynecol.* (2008) 32:923–8. 10.1002/uog.5405 18839405

[58] Zhang A Luo G Sun Y Chen T Pan S. Creation of high position fetal balloon atrial septoplasty for hypoplastic left heart syndrome and highly restrictive atrial septum: a case report and literature review. *J Interv Med.* (2020) 3:55–7. 10.1016/j.jimed.2020.01.009 34805908 PMC8562168

[59] Kalish BT Tworetzky W Benson CB Wilkins-Haug L Mizrahi-Arnaud A McElhinney DB Technical challenges of atrial septal stent placement in fetuses with hypoplastic left heart syndrome and intact atrial septum. *Catheter Cardiovasc Interv.* (2014) 84:77–85. 10.1002/ccd.25098 23804575

[60] Jantzen DW Moon-Grady AJ Morris SA Armstrong AK Berg C Dangel J Hypoplastic left heart syndrome with intact or restrictive atrial septum: a report from the international fetal cardiac intervention registry. *Circulation.* (2017) 136:1346–9. 28864444 10.1161/CIRCULATIONAHA.116.025873

[61] Tulzer A Arzt W Prandstetter C Tulzer G. Atrial septum stenting in a foetus with hypoplastic left heart syndrome and restrictive foramen ovale: an alternative to emergency atrioseptectomy in the newborn-a case report. *Eur Heart J Case Rep.* (2020) 4:1–4. 10.1093/ehjcr/ytaa005 32128498 PMC7047067

[62] Szwast A Tian Z McCann M Donaghue D Rychik J. Vasoreactive response to maternal hyperoxygenation in the fetus with hypoplastic left heart syndrome. *Circ Cardiovasc Imaging.* (2010) 3:172–8. 20044513 10.1161/CIRCIMAGING.109.848432 PMC3070268

[63] Lara DA Morris SA Maskatia SA Challman M Nguyen M Feagin DK Pilot study of chronic maternal hyperoxygenation and effect on aortic and mitral valve annular dimensions in fetuses with left heart hypoplasia. *Ultrasound Obstet Gynecol.* (2016) 48:365–72. 26700848 10.1002/uog.15846

[64] Szwast A Putt M Gaynor JW Licht DJ Rychik J. Cerebrovascular response to maternal hyperoxygenation in fetuses with hypoplastic left heart syndrome depends on gestational age and baseline cerebrovascular resistance. *Ultrasound Obstet Gynecol.* (2018) 52:473–8. 10.1002/uog.18919 28976608 PMC6719779

[65] Kohl T. Chronic intermittent materno-fetal hyperoxygenation in late gestation may improve on hypoplastic cardiovascular structures associated with cardiac malformations in human fetuses. *Pediatr Cardiol.* (2010) 31:250–63. 10.1007/s00246-009-9600-5 20024652 PMC2817075

[66] Enzensberger C Axt-Fliedner R Degenhardt J Kawecki A Tenzer A Kohl T Pulmonary vasoreactivity to materno-fetal hyperoxygenation testing in fetuses with hypoplastic left heart. *Ultraschall Med.* (2016) 37:195–200. 10.1055/s-0034-1385668 25607629

[67] Rasanen J Wood DC Debbs RH Cohen J Weiner S Huhta JC. Reactivity of the human fetal pulmonary circulation to maternal hyperoxygenation increases during the second half of pregnancy: a randomized study. *Circulation.* (1998) 97:257–62. 10.1161/01.cir.97.3.257 9462527

[68] Iwamoto HS Teitel DF Rudolph AM. Effect of birth-related events on metabolism in fetal sheep. *Pediatr Res.* (1991) 30:158–64. 1896261 10.1203/00006450-199108000-00007

[69] Rudolph AM. Maternal hyperoxygenation for the human fetus: should studies be curtailed? *Pediatr Res.* (2020) 87:630–3. 10.1038/s41390-019-0604-4 31600768

[70] Lee FT Marini D Seed M Sun L. Maternal hyperoxygenation in congenital heart disease. *Transl Pediatr.* (2021) 10:2197–209. 34584891 10.21037/tp-20-226 PMC8429855

[71] Schidlow DN Donofrio MT. Prenatal maternal hyperoxygenation testing and implications for critical care delivery planning among fetuses with congenital heart disease: early experience. *Am J Perinatol.* (2018) 35:16–23. 10.1055/s-0037-1603991 28746973

[72] Mardy C Kaplinski M Peng L Blumenfeld YJ Kwiatkowski DM Tacy TA Maternal hyperoxygenation testing in fetuses with hypoplastic left-heart syndrome: association with postnatal atrial septal restriction. *Fetal Diagn Ther.* (2021) 48:678–89. 10.1159/000519322 34673647

[73] Michelfelder E Gomez C Border W Gottliebson W Franklin C. Predictive value of fetal pulmonary venous flow patterns in identifying the need for atrial septoplasty in the newborn with hypoplastic left ventricle. *Circulation.* (2005) 112:2974–9. 10.1161/CIRCULATIONAHA.105.534180 16260632

[74] Taketazu M Barrea C Smallhorn JF Wilson GJ Hornberger LK. Intrauterine pulmonary venous flow and restrictive foramen ovale in fetal hypoplastic left heart syndrome. *J Am Coll Cardiol.* (2004) 43:1902–7. 10.1016/j.jacc.2004.01.033 15145119

[75] Better DJ Apfel HD Zidere V Allan LD. Pattern of pulmonary venous blood flow in the hypoplastic left heart syndrome in the fetus. *Heart.* (1999) 81:646–9. 10336926 10.1136/hrt.81.6.646 PMC1729071

